# Proportion of confirmed Lyme neuroborreliosis cases among patients with suspected early European Lyme neuroborreliosis

**DOI:** 10.21203/rs.3.rs-5231881/v1

**Published:** 2024-12-19

**Authors:** Katarina Ogrinc, Petra Bogovič, Tereza Rojko, Vera Maraspin, Eva Ružić-Sabljić, Andrej Kastrin, Klemen Strle, Gary P. Wormser, Franc Strle

**Affiliations:** University Medical Centre Ljubljana; University Medical Centre Ljubljana; University Medical Centre Ljubljana; University Medical Centre Ljubljana; University of Ljubljana; University of Ljubljana; Tufts University School of Medicine; New York Medical College; University Medical Centre Ljubljana

**Keywords:** Lyme neuroborreliosis, Bannwarth syndrome, Cranial neuritis, Borrelial meningitis, Intrathecal borrelial antibody synthesis, Borrelia culture from cerebrospinal fluid

## Abstract

**Purpose:**

To determine the frequency of confirmed Lyme neuroborreliosis (LNB) cases in adult patients with three different clinical presentations consistent with early LNB.

**Methods:**

Data were obtained through routine health care at the UMC Ljubljana, Slovenia from 2005–2022, using clinical pathways. The patients were classified into three groups: i) radicular pain of new onset (N = 332); or ii) involvement of cranial nerve(s) but without radicular pain (N = 997); or iii) erythema migrans (EM) skin lesion(s) in conjunction with symptoms suggestive of nervous system involvement but without either cranial nerve palsy or radicular pain (N = 240). The diagnosis of LNB considered the following variables: the presence of: 1) neurologic symptoms consistent with LNB (with no other obvious explanation); 2) cerebrospinal fluid (CSF) pleocytosis (> 5×10^6^ leukocytes/L); and 3) demonstration of intrathecal synthesis of borrelial antibodies, and/or cultivation of borrelia from CSF, and/or the presence of EM. Patients fulfilling only the first two criteria were interpreted as having possible LNB, while those who satisfied all three criteria were regarded as having confirmed LNB.

**Results:**

Of 1569 adult patients, 348 (22.2%) had confirmed LNB and 70 (4.5%) others had possible LNB. The proportion of confirmed LNB cases was the highest for patients with radicular pain (217/332, 65.4%), followed by the group with EM and neurologic symptoms (47/240, 19.6%), and those with cranial neuritis (84/997, 8.4%).

**Conclusion:**

Only 22% of patients evaluated had confirmed LNB. The proportion of confirmed LNB cases correlated with clinical presentation and was highest among patients with recent onset of radicular pain.

## INTRODUCTION

Lyme borreliosis (LB) is the most common tick-transmitted infection in the Northern Hemisphere. It is caused by *Borrelia burgdorferi* sensu lato (s. l.) [1]. The most common first sign of the disease, erythema migrans (EM), appears within days to weeks at the site of a tick bite [2]. Sometimes the infection disseminates, leading to development of multiple EM skin lesions and/or to involvement of non-cutaneous sites, such as the nervous system, joints or heart [3, 4].

Lyme neuroborreliosis (LNB) is the second most common clinical manifestation of disseminated LB in Europe after multiple EM and is reported to be principally caused by *Borrelia garinii* [5, 6]. Painful meningoradiculoneuritis (Bannwarth syndrome) is a typical clinical manifestation of early European LNB and is considered to be the most frequent manifestation of LNB in adult patients. Several other clinical manifestations, such as cranial neuritis, most often unilateral peripheral facial nerve palsy, and lymphocytic meningitis, have been recognized; however, the etiology of these conditions is diverse and the relative frequency of LNB as the cause of these particular conditions is not well understood [3, 4].

The objective of the present study was to evaluate the proportion of patients from the same geographic region with confirmed LNB, according to the different potential clinical presentations.

## PATIENTS AND METHODS

The approaches routinely used at the LB Outpatient Clinic for patients with suspected LNB were planned approximately 30 years ago. They included organized collection of data, clinical assessment and laboratory analyses that were performed in a similar manner throughout the time period evaluated (2005–2022) for the present analysis. The Clinic is open all year long for patient evaluations. Data were obtained through routine health care using clinical pathways and were analyzed retrospectively.

### Ethics.

This retrospective study was approved by the Medical Ethics Committee of the Ministry of Health of the Republic of Slovenia (0120–552/2023/3).

### Patients.

Research was conducted at the LB Outpatient Clinic of the Department of Infectious Diseases, UMC Ljubljana, where the vast majority of adult patients with suspected LNB from central part of Slovenia are assessed; only very rarely are such patients evaluated instead by the institutional Neurology Department. Patients ≥ 15 years of age (referred to herein as adult patients), referred by primary physicians due to clinically suspected early LNB during the period 2005–2022, who had: i) radicular pain of new onset, or ii) peripheral facial palsy (PFP) or involvement of other cranial nerves, but without radicular pain, or iii) solitary or multiple EM skin lesions accompanied by moderate to severe other symptoms suggestive of nervous system involvement (such as headache, vertigo, disturbances of sleep, concentration or memory disorders, paresthesias) and/or meningeal signs, and who consented to cerebrospinal fluid (CSF) examination, qualified for the study. All patients with CSF pleocytosis were tested for tick-borne encephalitis virus (TBEV) infection. Those with evidence of recent TBEV infection, defined by the presence of specific IgM and IgG antibodies in serum, were excluded. Demographic, epidemiologic and clinical data were obtained using a structured questionnaire.

### Laboratory Evaluation.

CSF was tested for cell counts and levels of total protein and glucose; a leukocyte count of > 5 × 10^6^ cells/L (pleocytosis) and a protein concentration of > 0.45 g/L were each considered to be abnormal findings. Immunoglobulin classes G and M (IgG and IgM, respectively) and albumin levels were determined in both serum and CSF by the immunonephelometric method using Siemens N antiserum.

### Serologic Evaluation.

Up to 2010, antibodies to *B. burgdorferi* s. l. in serum and CSF were determined by an indirect immunofluorescence assay (IFA) without absorption [7, 8]. Since 2011 an indirect chemiluminescence immunoassay (LIAISON^®^, Diasorin, Italy) has been used; results were interpreted according to the manufacturer’s instructions. On the basis of the IFA or LIAISON^®^ results, intrathecal borrelial antibody synthesis (ITBAS) was determined using the approach described by Reiber and Peter: antibody index values > 2.0 (IFA) or ≥ 1.5 (Liaison^®^) were interpreted as indicating intrathecal production of borrelial antibodies [9].

In patients with CSF pleocytosis serum IgM and IgG antibodies to TBEV were also determined, using Enzygnost^®^ Anti-TBEV (IgM, IgG) kits (Dade Behring Marburg GmbH, Marburg, Germany), according to the manufacturer’s instructions. The presence of specific IgM antibodies to TBEV in serum indicated a recent infection with the TBEV.

### Cultivation of B. burgdorferi s. l. from CSF.

Cultivation was performed as described previously [10–12].

#### Definitions

In the present study, the diagnosis of LNB was based on three variables: 1) presence of neurologic symptoms suggestive of LNB (with no other obvious explanation); 2) presence of CSF pleocytosis (> 5×10^6^ leukocytes/L); and 3) demonstration of borrelial infection by ITBAS IgM and/or IgG, and/or by cultivation of *Borreliae* from CSF, and/or by the presence of a concomitant EM skin lesion (or a reliable history of an EM skin lesion within the prior three months). Fulfillment of all three criteria was needed for the diagnosis of confirmed LNB, while patients who met only the first two criteria were interpreted to have possible LNB.

EM was defined as an expanding erythematous skin lesion, with or without central clearing, that developed days to a few weeks after a tick bite or exposure to ticks in an LB endemic geographic region and had a diameter ≥ 5 cm. If < 5 cm in diameter, a history of a tick bite at that site, a delay in the appearance of at least two days, and an expanding rash at the bite site were required for diagnosis of EM. Multiple EM skin lesions were defined by the presence of ≥ two skin lesions, at least one of which fulfilled the size criterion (≥ 5 cm) for a solitary EM [13].

### Statistical approaches.

Continuous variables were presented as median values and interquartile ranges (IQRs); discrete variables were denoted as counts and percentages along with 95% confidence intervals (CIs). Statistical comparisons among patient groups were evaluated using the Fisher exact test or the Wilcoxon rank-sum test. The Benjamini-Hochberg approach was employed for controlling the false discovery rate in multiple comparisons where applicable. A p value < 0.05 was considered to indicate statistical significance. All statistical analyses were conducted using R software [14].

## RESULTS

From October 2005 to December 2022, 1576 patients with clinically suspected early LNB, for whom CSF examination was performed, were examined at the LB Outpatient Clinic: 334 had radicular pain; 998 had PFP and/or involvement of another cranial nerve without radicular pain; and 244 had EM associated with symptoms suggestive of nervous system involvement but without radicular pain or cranial nerve palsy. The latter group represented a small subset (approximately 6%) of EM patients referred to our LB Outpatient Clinic, i.e. only those with pronounced symptoms suggestive of nervous system involvement [15].

**Figure 1:**
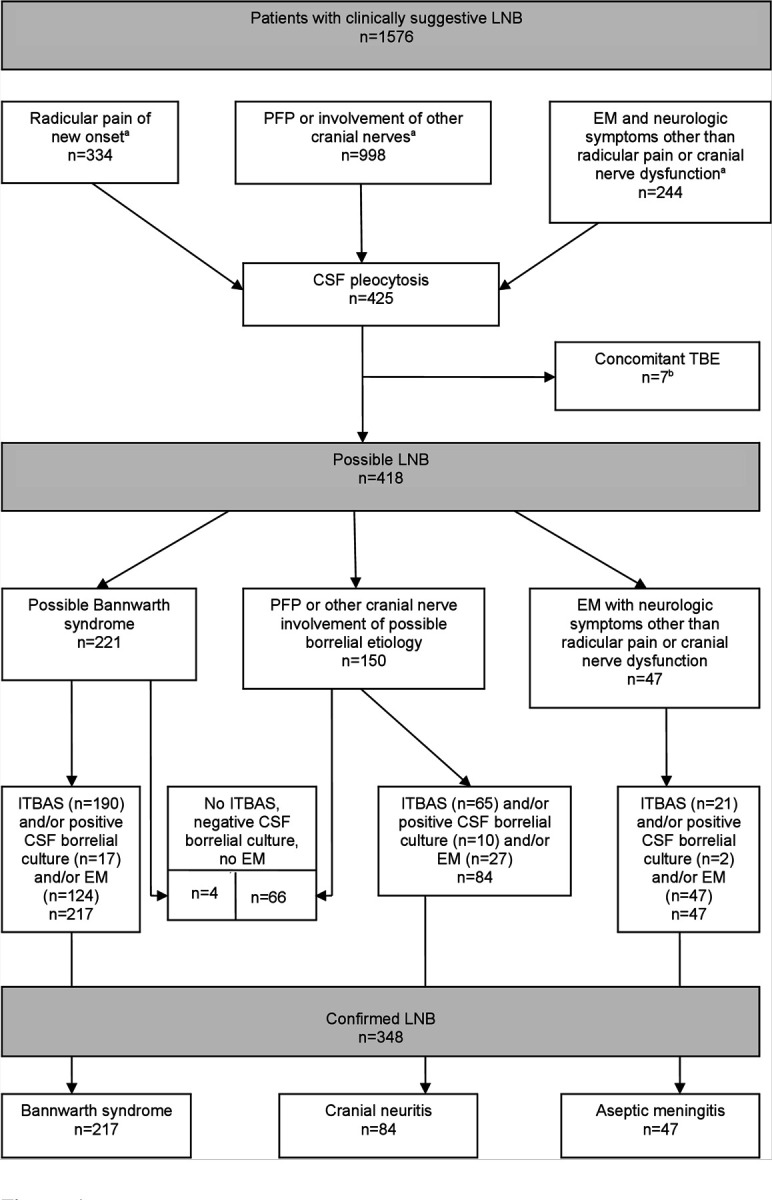
Flow chart of inclusion of patients with possible and confirmed Lyme neuroborreliosis at UMC Ljubljana in Slovenia, 2005–2022 ^a^Without other obvious reason. ^b^Excluded due to concomitant TBE (two patients with radicular pain of recent onset, one patient with cranial neuritis and four patients with erythema migrans and neurologic symptoms other than radicular pain or cranial nerve dysfunction). Abbreviations: LNB, Lyme neuroborreliosis; PFP, peripheral facial palsy; EM, erythema migrans; CSF, cerebrospinal fluid; TBE, tick-borne encephalitis; ITBAS, intrathecal borrelial antibody synthesis.

Of the 1576 patients referred for clinically suspected LNB, 425 (27.0%) had CSF pleocytosis. These 425 were tested for TBEV, and seven (1.6%) had serologic evidence of recent infection with TBEV and were excluded. Therefore, the diagnosis for LNB was assessed for 1569 patients, consisting of 332 patients with new onset of radicular pain, 997 patients with cranial nerve palsy without radicular pain, and 240 patients with EM and symptoms suggestive of nervous system involvement but without radicular pain or cranial nerve palsy (Fig. 1).

Of these 1569 patients, 418 (26.6%) had CSF pleocytosis; 348 (22.2%) patients fulfilled criteria for confirmed LNB, while 70 (4.5%) patients, with the absence of ITBAS, with a negative CSF culture for *Borrelia*, and without an EM skin lesion, were diagnosed as having possible LNB. Among the 348 patients with confirmed LNB, 217 (62.4%) had Bannwarth syndrome, 84 (24.1%) had cranial neuritis without radicular pain and 47 (13.5%) had CSF pleocytosis without radicular pain or cranial neuritis (Fig. 1).

Therefore, of the 1569 evaluable patients with suspected early LNB, the proportion with confirmed LNB was significantly (p<0.001) higher in the group with new onset of radicular pain (217/332, 65.4%) than in either of the two groups without radicular pain: 47/240 (19.6%) in patients with EM and neurologic symptoms, and 84/997 (8.4%) in patients with cranial neuritis. The flow chart of the basic diagnostic categorization is shown in the Fig. 1, with more detailed evidence for the presence of borrelial infection in patients, who had a clinical presentation compatible with LNB associated with CSF pleocytosis, shown in the Table 1.

Borreliae that cause LB are transmitted by tick bites. Since tick activity has a seasonal pattern, the occurrence of LNB is expected to have a corresponding seasonal pattern, however, with a time delay of a few days to several weeks. As shown in Table 2, the month of occurrence for the first (neurologic) symptoms/signs for the 70 patients diagnosed with possible LNB and for the 348 patients who fulfilled criteria for confirmed LNB differed, while the subgroups with the 3 different clinical presentations within the group of confirmed LNB had a similar monthly distribution (Table 3). Patients with confirmed LNB showed a marked seasonal distribution of disease onset, while patients with possible LNB showed a more even distribution by month. Namely, in 299/348 (85.9%) patients with confirmed LNB, neurologic symptoms started in the warm period of the year between May and October, whereas among patients with possible LNB, the proportion in the same 6-month period was only 34/70 (48.6%); p<0.001. In contrast, during the cold half of the year (between November and April), the proportion with disease onset was lower among patients with confirmed LNB than among those with possible LNB. As shown in Table 2, this was true not only for the entire 6-month period (49/348, 14.1% vs 36/70, 51.4%; p<0.001) but also for individual months (November and March).

As expected, of the three criteria used to establish borrelial infection in patients with suspected LNB in the present study, ITBAS was the most common. It is of interest that the ITBAS criterion was fulfilled in the majority of patients with Bannwarth syndrome (86%) but in less than one-half of patients belonging to the two LNB groups without radicular pain: in 43% of those with cranial neuritis and in 45% of those with CSF pleocytosis in conjunction with EM and neurologic symptoms, but without either radicular pain or cranial neuropathy. However, since the clinical groups differed significantly in the duration of symptoms (Table 1) and since the intrathecal production of *Borrelia* antibodies may not appear until several weeks after the onset of neurologic involvement, this difference might be due to the longer duration of illness in patients with Bannwarth syndrome at the time of evaluation than in the other two clinical groups. The comparison of the duration of illness between ITBAS positive and ITBAS negative patients showed that the duration of illness was longer in ITBAS positive patients for the whole study group (22 vs. 11 days, p<0.001), for patients with Bannwarth syndrome (30 vs. 11 days, p<0.001) and for patients with cranial neuritis (18 vs. 7 days, p=0.04), while in EM patients with CSF pleocytosis but without radicular pain or cranial nerve involvement, the duration of illness differed by an average of 7 days, but this difference was not statistically significant (14 vs. 21 days, p=0.55) (Tables 4 and 5).

## DISCUSSION

The aim of the present study was to assess the proportion of confirmed LNB among patients with clinically suspected early LNB, and to evaluate and compare the proportion of confirmed LNB for three different clinical presentations, i.e., for patients who present with radicular pain of new onset, those with PFP and/or involvement of another cranial nerve without radicular pain, and those with EM associated with symptoms suggestive of nervous system involvement but without either radicular pain or cranial nerve palsy. We chose these three groups to evaluate because they represent the most common clinical manifestations of LNB in adult patients in Europe [3, 16].

The analysis used in the current study is based on the same reasoning as is used in everyday clinical practice in patients with a reasonable clinical suspicion of LNB without obvious other causes of nervous system involvement. Since the diagnosis of LNB in accordance with the European Federation of Neurological Societies (EFNS) criteria [17] requires a CSF examination, and since basic CSF results (leukocyte count) are available relatively quickly (in <1 hour) and much earlier than the results of serologic tests for *Borrelia* or even the results of *Borrelia* cultures, we used the corresponding “natural clinical” sequence as shown in the Fig. 1: 1. Clinical suspicion of LNB without obvious other causes of nervous system involvement; 2. Confirmation of inflammation of the central nervous system (demonstration of CSF pleocytosis); and 3. Evidence indicating that the inflammation is caused by *Borrelia*.

The main findings of the present study were the pronounced differences in the probability for confirmed LNB among the different presentations of clinically suspected LNB evaluated. The majority (63.5%) of the 1569 patients from Slovenia evaluated in this study presented with PFP and/or involvement of another cranial nerve without radicular pain, 21.2% had radicular pain of new onset, while 15.3% had EM and neurologic symptoms without radicular pain or cranial nerve palsy. Of these 1569 patients, 348 (22.2%) fulfilled the criteria for confirmed LNB: 217 had Bannwarth syndrome, 84 had cranial neuritis with CSF pleocytosis and 47 had CSF pleocytosis with EM but without radicular pain or cranial neuritis. Although these findings corroborate previous reports that Bannwarth syndrome is the most frequent manifestation of early European LNB in adults [6, 18–20], it should be mentioned that in the present study, patients were predominantly referred to us by primary care physicians. Although the large majority of patients with suspected LNB are referred to our LB Outpatient Clinic and only a few instead to neurologists, the proportions of referred patients with individual clinical manifestations might differ from the corresponding proportions of LNB clinical manifestations in other health care settings.

Our study demonstrated that the proportion of confirmed early LNB substantively differed when comparing three distinct clinical presentations. Although among patients referred for suspected LNB those with cranial nerve involvement were the most common, in the group with confirmed LNB the number of patients with Bannwarth syndrome (clinically characterized with radicular pain) was substantially higher compared with those who presented with cranial neuritis, and compared with those who had EM associated neurologic symptoms and CSF pleocytosis but without cranial neuritis and/or radicular pain (aseptic meningitis). The reason for this was the different proportions of confirmed LNB among the 3 clinically suspected LNB groups: the proportion was highest in the group with new onset of radicular pain (217/332, 65.4%), which was 6.1-times higher than in patients without radicular pain (131/1237, 10.6%; p<0.001) - i.e., 3.3-times higher compared to the group with EM associated neurologic symptoms but without cranial neuritis and/or radicular pain (47/240, 19.6%; p<0.001), and 7.8-times higher than in patients with cranial neuritis (84/997, 8.4%; p<0.001).

Of the 1569 patients evaluated, 348 (22.2%) fulfilled the criteria for confirmed LNB, whereas 1221 did not. Of the 1221 patients who did not meet criteria for confirmed LNB, 1151 (94.3%) patients had a normal CSF leukocyte count, while 70 (5.7%) patients had CSF pleocytosis but did not fulfill at least one of the three requirements for proof of *Borrelia* infection (ITBAS, isolation of borrelia from CSF, or presence of EM). The latter group of 70 patients comprised four patients with new onset of radicular pain, and 66 patients with cranial neuritis without radicular pain. The fraction of patients who had CSF pleocytosis but did not fulfill at least one of the three criteria to confirm LNB was 4 of 221 (1.8%) patients with radicular pain, 66 of 150 (44%) patients with cranial neuritis without radicular pain, and none of 47 EM patients with neurologic symptoms other than radicular pain or cranial nerve involvement (Fig. 1). Thus, the finding of CSF pleocytosis in a patient with radicular pain of new onset indicates a very high probability for confirmed LNB, while the corresponding finding in a patient with cranial nerve involvement has a much lower predictive value. Of interest, but not surprisingly according to some European reports on LNB [21–23], the likelihood of confirmation of the diagnosis of confirmed LNB was associated with month of onset of the clinical illness. Onset in the 6-month period from May through October was statistically significantly higher for confirmed LNB compared with possible LNB cases, p<0.001. The more even distribution of disease onset by month in patients with possible LNB indicates a less close temporal association with tick bites and suggests that this group includes persons without LNB.

Key strengths of our research approach are the large cohorts of well-defined patients with distinct clinical presentations suggestive of early LNB; the origin of patients from the same geographic region that enables an identical LB epidemiologic pattern for different clinical presentations; a comprehensive and standardized diagnostic approach over the 17 year study period, and an evaluation for all patients that included a clinical examination, an examination of CSF, a calculation of ITBAS, and a *Borrelia* culture of CSF (culture was performed in 397/418 (95%) possible or confirmed LNB cases); and the use of standardized LNB case definitions. In addition, the month of onset of symptoms was evaluated.

However, in addition to a potential referral bias, a limitation of this study is that it is based on patients at least 15 years of age and thus the findings, although relevant, may not be completely applicable to younger age children. Moreover, although we believe that our findings are relevant to the other European regions endemic for LB, it is not clear how such findings may relate to patients with LNB in North America, where the disease is almost exclusively due to the borrelial species *B. burgdorferi*, and borrelial meningoradiculoneuritis is rare [24–26]. Another limitation of our study is that CSF *Borrelia* culture was only performed for 95% of the patients. Culturing Lyme borrelia from CSF has low sensitivity (in the present study *Borrelia* was isolated from CSF from only 29/397 (7.3%) patients with CSF pleocytosis), is a test that is not usually available to clinicians, and requires a considerable work-burden with a substantial delay in getting the test results. However, culture positivity is highly specific and offers the opportunity for genetic analysis of the recovered isolates of *B. burgdorferi* s. l. Of potential relevance, PCR for detection of *Borrelia* in CSF was not performed; it is not a part of routine testing for LNB at our institution, since it has a relatively low sensitivity in CSF [27, 28]. However, future studies might consider adding PCR testing. In addition, testing for other pathogens that cause CSF pleocytosis besides TBEV was not done (e.g., Herpes simplex virus (HSV), varicella zoster virus (VZV), enteroviruses, and *Treponema pallidum*). However, a study on the etiology of peripheral facial palsy performed in the same geographical region revealed that likelihood of finding an etiology other than *Borrelia* was negligible: for example, of 364 patients for whom molecular testing for HSV-1, HSV-2, and VZV DNA was performed in CSF, none tested positive for HSV-1, none for HSV-2, and only 1 for VZV DNA [29].

In conclusion, although in our study nearly 3-times more patients with cranial neuritis than patients with radicular pain of new onset were included, the study findings corroborated previous reports that in a LB endemic region in Europe, such as Slovenia, borrelial meningoradiculoneuritis (Bannwarth syndrome) is the most frequent clinical manifestation of early LNB in adult patients [6, 18–20]. The main reason for this finding were the pronounced differences in the probability for confirmed LNB among the different presentations of clinically suspected LNB. Patients with suspected LNB who presented with a new onset of radicular pain had a high (65%) probability for LNB, while the corresponding probability was more than 6-times lower for those who presented without radicular pain, - i.e., more than 3-times lower in patients with neurologic symptoms associated with EM but without cranial neuritis (20%), and nearly 8-times lower in patients who presented with cranial neuritis (8%). Seasonality is also a pertinent variable in terms of the likelihood of diagnosing confirmed cases of LNB.

## Figures and Tables

**Table 1.** Demonstration of borrelial infection in patients who had a clinical presentation compatible with Lyme neuroborreliosis associated with cerebrospinal fluid pleocytosis (N=418)

Criterion		Radicular pain	No radicular pain	
		Bannwarth syndrome n = 221	Cranial neuritis n = 150	Aseptic meningitis n = 47
ITBAS	Present	190 (86.0)	65 (43.3)	21 (44.7)
	Sole criterion	87 (39.4)	47 (31.3)	0
Isolation of borrelia from CSF	Present	17^a^/210 (8.1)	10^b^/145 (6.9)	2^c^/46 (4.3)
	Sole criterion	0	3 (2.0)	0
Erythema migrans	Present	124^d^ (56.1)	27^e^ (18.0)	47^f^ (100)
	Sole criterion	20 (9.0)	10 (6.7)	24 (51.1)
Duration of illness (days) (IQR)^g,h^		25 (14–45)	10 (5–21)	18 (8–35)
Duration of neurologic symptoms (days) (IQR)^g,i^		23 (14–42)	8 (5–21)	10 (7–30)

Data are frequencies (%) or median (IQR).

Abbreviations: ITBAS, intrathecal borrelial antibody synthesis; CSF, cerebrospinal fluid; IQR, interquartile range.

a 11/17 (65%) patients had positive ITBAS, 12/17 (71%) patients had erythema migrans.

b 5/10 (50%) patients had positive ITBAS, 4/10 (40%) patients had erythema migrans.

c 0/2 patients had positive ITBAS, both patients had erythema migrans.

d 98/124 (79%) patients had positive ITBAS, 12/124 (10%) patients had positive CSF borrelial culture.

e 15/27 (56%) patients had ITBAS, 4/27 (15%) patients had positive CSF borrelial culture.

f 21/47 (45%) patients had ITBAS, 2/46 (4%) had positive CSF borrelial culture.

g Up to time of enrollment.

h Duration of illness in patients with Bannwarth syndrome was significantly longer than in patients with cranial neuritis (p<0.001), or lymphocytic meningitis without radicular pain (p=0.04).

i Duration of neurologic symptoms in patients with Bannwarth syndrome was significantly longer than in patients with cranial neuritis (p<0.001) or lymphocytic meningitis without radicular pain (p<0.001).

**Table 2.** Seasonality of the beginning of neurologic symptoms in confirmed and possible Lyme neuroborreliosis (n=418)

	Confirmed LNB n = 348	Possible LNB n = 70	p	p_adj_
January	6 (1.7)	5 (7.1)	**0.028**	0.091
February	2 (0.6)	2 (2.9)	0.137	0.222
March	5 (1.4)	6 (8.6)	**0.006**	**0.037**
April	6 (1.7)	4 (5.7)	0.076	0.164
May	35 (10.1)	5 (7.1)	0.655	0.655
June	64 (18.4)	7 (10.0)	0.161	0.233
July	77 (22.1)	9 (12.9)	0.192	0.249
August	63 (18.1)	8 (11.4)	0.295	0.348
September	41 (11.8)	3 (4.3)	0.125	0.222
October	19 (5.5)	2 (2.9)	0.551	0.596
November	15 (4.3)	11 (15.7)	**0.003**	**0.037**
December	15 (4.3)	8 (11.4)	**0.044**	0.114
May–October	299 (85.9)	34 (48.6)	**<0.001**	**0.046**

Data are frequencies (%). Boldface is used for statistically significant differences.

Abbreviations: LNB, Lyme neuroborreliosis; p_adj_, adjusted raw p value for multiple comparisons.

**Table 3.** Seasonality of the beginning of neurologic symptoms in patients with different manifestations of confirmed Lyme neuroborreliosis (n=348)

	Bannwarth syndrome n = 217	Cranial neuritis n = 84	Aseptic meningitis n = 47	p_1_	p_2_	p_3_
January	3 (1.4)	2 (2.4)	1 (2.1)	0.623/0.899	0.548/>0.994	0.999/0.999
February	0	2 (2.4)	0	0.080/0.519	0.999/0.999	0.540/0.999
March	2 (0.9)	2 (2.4)	1 (2.1)	0.316/0.741	0.450/0.994	0.999/0.999
April	5 (2.3)	1 (1.2)	0	0.999/0.999	0.591/0.994	0.999/0.999
May	20 (9.2)	9 (10.7)	6 (12.8)	0.829/0.999	0.593/0.994	0.781/0.999
June	39 (18.0)	16 (19.0)	9 (19.1)	0.871/0.999	0.840/0.994	0.999/0.999
July	54 (24.9)	16 (19.0)	7 (14.9)	0.456/0.741	0.259/0.994	0.813/0.999
August	43 (19.8)	12 (14.3)	8 (17.0)	0.411/0.741	0.841/0.994	0.804/0.999
September	22 (10.1)	12 (14.3)	7 (14.9)	0.424/0.741	0.448/0.994	0.999/0.999
October	10 (4.6)	6 (7.1)	3 (6.4)	0.403/0.741	0.710/0.994	0.999/0.999
November	7 (3.2)	6 (7.1)	2 (4.3)	0.207/0.741	0.666/0.994	0.712/0.999
December	12 (5.5)	0	3 (6.4)	**0.041**/0.519	0.737/0.994	0.050/0.650
May–October	188 (86.6)	71 (84.5)	40 (85.1)	0.925/0.999	0.999/0.999	0.999/0.999

Data are frequencies (%). P values (p_1_–p_3_) are presented as raw p value/adjusted raw p value for multiple comparisons. Boldface is used for statistically significant difference.

P_1_: Bannwarth syndrome vs. cranial neuritis.

P_2_: Bannwarth syndrome vs. aseptic meningitis.

P_3_: Cranial neuritis vs. aseptic meningitis.

**Table 4.** Duration of illness and neurologic symptoms in ITBAS+ and ITBAS− patients with different clinical manifestations of suspected Lyme neuroborreliosis associated with cerebrospinal fluid pleocytosis (n=418)

	ITBAS+	ITBAS−	p	p_adj_
All patients, n = 418	n = 276	n = 142		
Duration of illness (days)	22 (14–40)	10 (6–21)	**<0.001**	**<0.001**
Duration of neurologic symptoms (days)	21 (12–40)	9 (5–18)	**<0.001**	**<0.001**
Bannwarth syndrome, n = 221	n = 190	n = 31		
Duration of illness (days)	30 (14–45)	11 (7–26)	**<0.001**	**0.001**
Duration of neurologic symptoms (days)	26 (14–45)	11 (7–21)	**<0.001**	**0.001**
Cranial neuritis, n = 150	n = 65	n = 85		
Duration of illness (days)	18 (7–29)	8 (5–16)	**0.020**	**0.031**
Duration of neurologic symptoms (days)	14 (4–23)	7 (5–15)	0.168	0.223
Erythema migrans + aseptic meningitis, n = 47	n = 21	n = 26		
Duration of illness (days)	14 (8–30)	21 (10–45)	0.548	0.627
Duration of neurologic symptoms (days)	10 (7–23)	13 (5–30)	0.833	0.833

Data are median (IQR). Boldface is used for statistically significant differences.

Abbreviations: ITBAS+, positive intrathecal borrelial antibody synthesis; ITBAS−, negative intrathecal borrelial antibody synthesis; p_adj_, adjusted raw p value for multiple comparisons.

**Table 5.** Duration of illness and neurologic symptoms in ITBAS+ and ITBAS− patients with confirmed Lyme neuroborreliosis (n=348)

	ITBAS+	ITBAS−	p	p_adj_
All patients, n = 348	n = 276	n = 72		
Duration of illness (days)	22 (14–40)	11 (7–30)	**<0.001**	**<0.001**
Duration of neurologic symptoms (days)	21 (12–40)	10 (7–21)	**<0.001**	**<0.001**
Bannwarth syndrome, n = 217	n = 190	n = 27		
Duration of illness (days)	30 (14–45)	11 (7–18)	**<0.001**	**<0.001**
Duration of neurologic symptoms (days)	26 (14–45)	11 (7–18)	**<0.001**	**<0.001**
Cranial neuritis, n = 84	n = 65	n = 19		
Duration of illness (days)	18 (7–29)	7 (4–10)	**0.036**	0.057
Duration of neurologic symptoms (days)	14 (4–23)	7 (4–10)	0.176	0.235
Erythema migrans + aseptic meningitis, n = 47	n = 21	n = 26		
Duration of illness (days)	14 (8–30)	21 (10–45)	0.548	0.627
Duration of neurologic symptoms (days)	10 (7–23)	13 (5–30)	0.833	0.833

Data are median (IQR). Boldface is used for statistically significant differences.

Abbreviations: ITBAS+, positive intrathecal borrelial antibody synthesis; ITBAS−, negative intrathecal borrelial antibody synthesis; p_adj_, adjusted raw p value for multiple comparisons.

## Data Availability

All relevant data are presented in the manuscript. More detailed data can be provided by the authors upon reasonable request.
